# Non-contact respiratory rate monitoring using thermal and visible imaging: a pilot study on neonates

**DOI:** 10.1007/s10877-022-00945-8

**Published:** 2022-12-04

**Authors:** Lalit Maurya, Reyer Zwiggelaar, Deepak Chawla, Prasant Mahapatra

**Affiliations:** 1 Academy of Scientific and Innovative Research (AcSIR), Ghaziabad, 201002 India; 2 CSIR-Central Scientific Instruments Organisation (CSIR-CSIO), Sector 30-C, Chandigarh, 160030 India; 3 Department of Computer Science, Aberystwyth University, Ceredigion, SY23 3DB UK; 4 Department of Neonatology, Government Medical College & Hospital (GMCH), Chandigarh, 160030 India

**Keywords:** Non-contact, Respiratory rate, Remote monitoring, Thermal imaging, Neonates

## Abstract

Respiratory rate (RR) monitoring is essential in neonatal intensive care units. Despite its importance, RR is still monitored intermittently by manual counting instead of continuous monitoring due to the risk of skin damage with prolonged use of contact electrodes in preterm neonates and false signals due to displacement of electrodes. Thermal imaging has recently gained significance as a non-contact method for RR detection because of its many advantages. However, due to the lack of information in thermal images, the selection and tracking of the region of interest (ROI) in thermal images for neonates are challenging. This paper presents the integration of visible (RGB) and thermal (T) image sequences for the selection and tracking of ROI for breathing rate extraction. The deep-learning based tracking-by-detection approach is employed to detect the ROI in the RGB images, and it is mapped to the thermal images using the RGB-T image registration. The mapped ROI in thermal spectrum sequences gives the respiratory rate. The study was conducted first on healthy adults in different modes, including steady, motion, talking, and variable respiratory order. Subsequently, the method is tested on neonates in a clinical settings. The findings have been validated with a contact-based reference method.The average absolute error between the proposed and belt-based contact method in healthy adults reached 0.1 bpm and for more challenging conditions was approximately 1.5 bpm and 1.8 bpm, respectively. In the case of neonates, the average error is 1.5 bpm, which are promising results. The Bland–Altman analysis showed a good agreement of estimated RR with the reference method RR and this pilot study provided the evidence of using the proposed approach as a contactless method for the respiratory rate detection of neonates in clinical settings.

**Supplementary Information:**

The online version contains supplementary material available at 10.1007/s10877-022-00945-8.

## Introduction

The first month after birth is crucial for the newborn infant’s survival, especially when born prematurely. During the first month after birth, vital signs must be continuously monitored. The respiratory rate is a vital indicator in neonatal intensive care units (NICU) [1]. Respiratory diseases are one of the leading causes of morbidity in neonates, and the incidence of these diseases is higher among preterm neonates [2, 3]. Many preterm neonates need admission and monitoring in special care neonatal units. Studies have shown that there is an increment in the number of neonatal admission due to respiratory distress [4]. Detection and assessment of respiratory dysfunction at an early stage enable appropriate medical treatment, and respiratory monitoring saves the lives of newborns [5].

The respiratory rate (RR) measurement involves the counting of breathing cycles per minute and is generally indicated by bpm (breath per minute). The breathing cycle is composed of inspiration and expiration, which are physiological phenomena used for the intake of oxygenated air into the lungs and the release of carbon dioxide-rich air to the environment, respectively. Generally, the intake air temperature is lower than the exhaled air at room temperature. The range of RR in healthy adults varies from 12 to 20 bpm whereas newborn infants breathe at a faster rate and normal RR in neonates varies from 30 to 60 bpm [6, 7]. The literature has evidence that despite the importance of continuous respiration monitoring benefits, it is usually ignored in primary care. Physician and nursing staff usually measure the respiratory rate manually by counting abdominal and chest movements [1, 8].

Two methods based on a wearable sensor are predominantly used for RR measurement. One is measuring the changes in impedance due to the chest and abdomen movements. The second is indirectly measured by a pulse oximeter. To measure the impedance changes, a small electrical signal is passed through the adhesive skin electrodes. The placement and removal of these adhesive skin sensors (electrodes) and the presence of all sensors cause discomfort and can even be painful [9].

Neonates with a gestational age of less than 28 weeks are more prone to suffering from epidermal stripping due to the use of adhesive sensors [10]. Premature infants, particularly those with an underdeveloped central nervous system, are extremely susceptible to external stimuli. Another set of problems, including motion artefacts, sensor dislocation, calibration shift, false connections, and the possibility of infection are associated with adhesive sensors. Motion artefacts due to the dislocation of the sensor are problematic when attempting continuous respiratory rate detection in neonates. Also, the chest impedance presents a limitation when detecting episodes of apnea [11]. Hence, an alternative, non-contact method would resolve these issues for the monitoring of respiration rate to provide neonatal care in both intensive care centres and homes.

This study aims to develop an approach for automatic region of interest (ROI) selection based on a contactless respiration rate (RR) measurement. The proposed method integrates visible and thermal imaging to estimate the RR. The visible image is used to find the facial landmarks for the ROI selection and tracking, while the thermal image is utilized to extract the respiratory signals. The contributions in this work are:

- The registration algorithm provides a linear mapping between thermal imaging and visible imaging so that the extracted facial landmarks in the visible domain are correctly mapped to the corresponding thermal image.
- A deep learning-based approach is employed for ROI selection and tracking.
- For the proof of concept, first, we applied the proposed method among healthy adults in a lab setting. Then, the method was tested on neonates in a clinical setting.

## Related work

In recent years, significant efforts have been made to develop a non-contact-based method for RR monitoring to overcome the drawbacks of the contact-based methods. Laser Doppler, radio frequency, and ultra-wideband impulse radio have been investigated for the extraction of respiration rate. Scalice et al. [12, 13] used a laser Doppler vibrometer in a contactless manner to detect abdominal movement for RR calculation; however, this method risks exposing neonates' eyes to laser light and relies on surface reflectance. Kim et al. [14] used impulse radio ultra-wideband on neonates to estimate the breathing cycle. The results were promising and correlated well with the contact-based impedance pneumography. The method has the benefit of being able to detect the signal under challenging conditions, such as a baby being covered with blankets or garments, and even in darkness, however, it was found to be sensitive to the newborn infant’s motion. Another approach by Khaemphukhiao et al. [15] used a radio frequency for the detection of RR, but the method was not tested in a clinical setting. The major concern with the radar-based method is the use of electromagnetic waves. Radiation exposure has a negative impact on the health of neonates and may sometimes cause adverse effects such as nervous system disorder [16]. As a result, researchers have taken an interest in the development of methodologies that employs passive modalities, such as visible cameras and infrared thermography. In addition, the camera-based methodology supports remote measurement and a large field of view (FOV), which adds the possibility of detecting the respiratory signals of multiple people. The visible camera has been used to find the RR either by using the concept of motion-based or reflectance photoplethysmography (PPG) based. The motion-based technique works on the basis of the detection of subtle changes in the chest wall, whereas the PPG-based technique detects the change in optical characteristics of the red, green, or blue spectrum of light transmitted or reflected by human skin. The motion-based methods have challenges in separating the breathing-induced movements from the movements not related to the breathing process.

Gastel et al. [17] presented a method of extracting the breathing signal from breathing-induced skin colour changes by exploiting the spatial redundancy in both visible and infrared lighting conditions. The method was tested on the NICU and achieved a correlation of 0.87. Cobos et al. [18] used the skin colour variation in a particular area of the newborn baby’s diaphragm to find the heart rate and breathing rate. Sun et al. [19] compared the conventional optical flow and deep learning-based flow to calculate the breathing-induced motion matrix. The breathing signal was obtained through motion factorization and compared with the signal extracted via chest impedance in the NICU. Villarroel et al. [20] proposed a multi-task convolutional neural network (CNN) for the segmentation of skin or non-skin automatically and estimated the vital signs from the segmented skin only when the newborn was in the field of view. The clinical aspect of the newborn data sample were studied in detail. Additionally, it performed well in low-light conditions. However, it has been suggested that more research is needed for dark-skinned subjects and dark ambient conditions. Further, Khanam et al. [21] used a CNN for the ROI selection and noise-assisted signal decomposition to supress the noise in the extracted respiratory signal.

Abbas et al. [22] proposed an approach using thermal imaging to extract respiration signals from premature infants. The nostril region was selected as the ROI and the nasal airflow temperature variation was used to calculate the RR. Klaessens et al. [23] used a visible colour camera to find the heart rate and a thermal camera to obtain the respiration rate and validated it using an ECG-based technique. Pereira et al. [24] used a high definition infrared camera and particle filter-based tracking on high-resolution infrared frames to find the RR in newborns. Furthermore, a Butterworth filter was applied to obtain a smoothed respiratory signal for RR monitoring purpose. The ROI selection for this work was manual. For a similar objective in the case of newborn babies, Pereira et al. [25] developed an automatically ROI selection based ‘black-box’ algorithm in which the movement of the grid boxes with a high signal to noise ratio is fused on a probability basis to extract the respiration movement. However, this methodology works efficiently even in frontal-view cases, which was the limitation in the previous study [24], as there is no need for nostril detection in this methodology. Lorato et al. [26] proposed an automatic ROI selection-based algorithm by merging three low resolution thermal camera views to extract the breathing signal from the pixels that have respiration motion or flow. Besides the benefit of not depending on facial landmark detection, the algorithm dealt with the problems of significant motion of infants and the presence of another person in the FOV. Further, Lorato et al. [27] addressed the problems of more challenging conditions such as head and limb movement and also the motion caused by non-nutritive sucking by motion detection and optimizing the motion that hides the respiration information.

## Proposed methodology

**Fig. 1:**
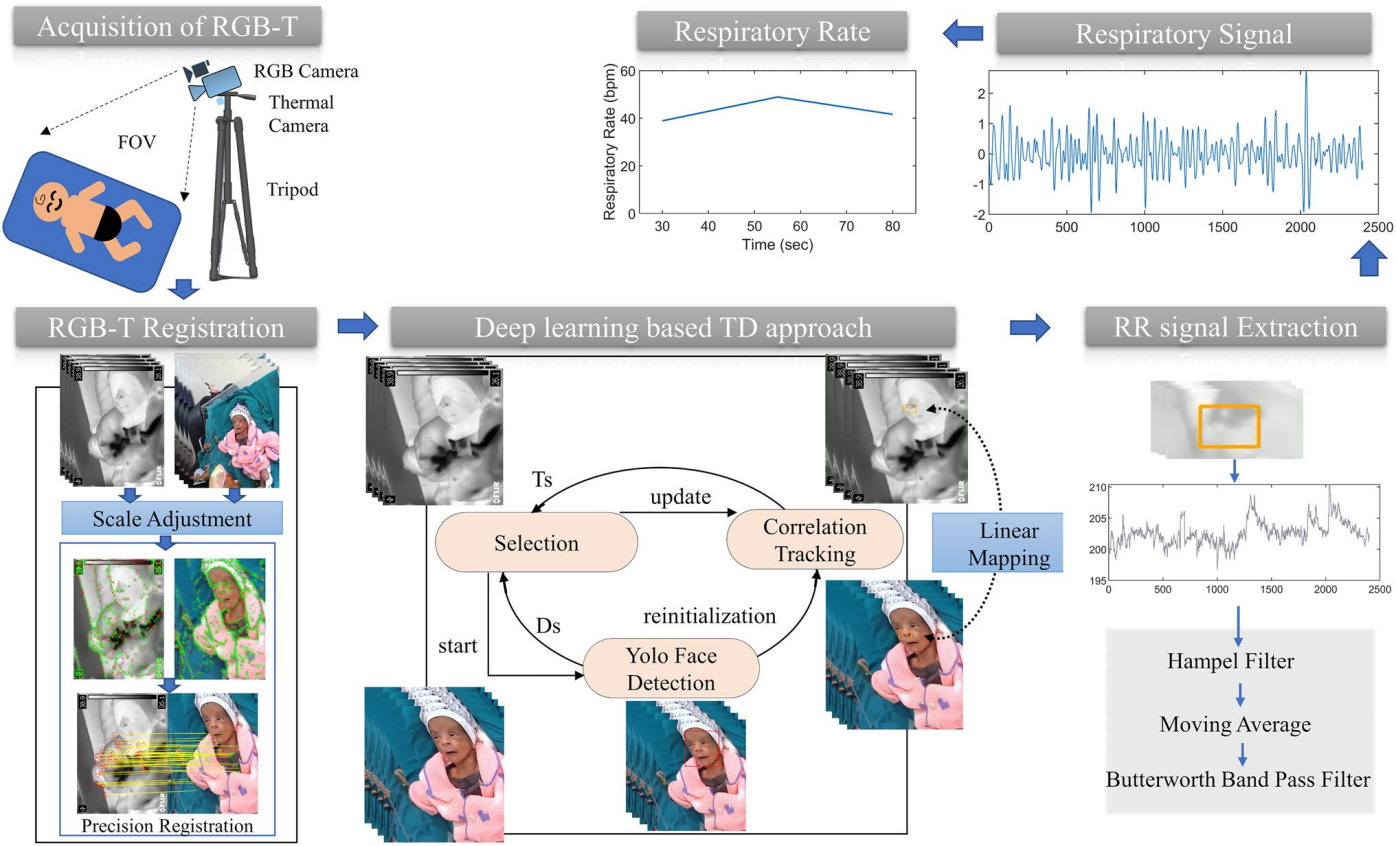
The flowchart depicting the proposed method's steps. The RGB-T video dataset is collected by the dual camera setup. The RGB-T registration gives the transformation matrix to provide the linear mapping. The deep learning-based TD approach performs the ROI selection and tracking in RGB images and by using transformation matrix maps in the thermal spectrum images. Some filtering approaches are utilized to extract the respiratory signal from the raw signal

In this work, the visible image sequences are integrated with the thermal image sequences for the breathing rate detection. For the integration of visible and thermal images (termed RGB-T) spectrum data is collected using a dual imaging setup and aligned by the RGB-T image registration. Visible images are used to detect and track the ROI, and thermal images are used to extract the respiratory signal from the selected ROI. The flowchart of the methodology is depicted in Fig. 1, and each step is elaborated in detail below.

### Acquisition of RGB-T

The integration of thermal and visible image sequences captured by the visible and thermal cameras gives the benefits of using the complementary features of RGB and thermal images. The RGB-T dataset is acquired by visible and thermal cameras simultaneously. To capture the same field of view with both cameras, they should be positioned parallel to one another and separated by a modest distance, as per the pinhole camera paradigm. The details of camera specification and configuration are discussed in the experimental subsection. The acquisition is triggered by the custom acquisition software with a zero-lapse time.

### RGB-T image registration

The two modalities have different principles of imaging. The visible camera captures the RGB image, which captures the object's information in the visible spectrum, while the thermal camera captures the object’s information in the infrared spectrum. In the integration of RGB-T spectrum data, the RGB spectrum has more information than the thermal spectrum, and the thermal image adds a meaningful band of information for determining the temperature distribution of objects. Due to the different modalities and configurations of cameras, the alignment of both types of images is required. By utilizing the RGB-T registration, the frames from the visible camera are aligned to the thermal frames. Traditionally, both cameras are usually fixed, and calibration is performed to obtain the aligned images. The scale and translation parameters obtained by performing the calibration were used to obtain the transformation matrix for alignment. This type of transformation depends on the camera parameters, and parameters vary according to the camera and distance from the object. To overcome this problem, we used the automatic two-phase registration method. First, the scale of large-sized visible images is adjusted to align them with the thermal image coordinate. After that, the precise registration is employed to tune the alignment of RGB-T pairs. A calibration rig is used for the scale adjustment method to automatically detect correspondence points in both RGB and thermal image pairs. The calibration rig is a square-cut checkerbox constructed from a material that is an inefficient heat conductor. The calibration rig is used during the initial phase of recording, and the person holds the rig during the initial recording in front of his body to emphasize the good contrast. More details about the calibration-based method can be found in previous work [28]. Due to a large difference in resolution between RGB and thermal images, there is a significant scale disparity between RGB and thermal image pairings. The scale adjustment steps overcome the large-scale disparity between RGB and thermal image pairs. In case of newborn infants data acquisition, the calibration is performed during setup. Although the scale adjustments aligned the RGB image sequences, there might be some misalignment caused by the selection of limited number of correspondence points from a small region. After the scale adjustment, the alignment is refined using a phase congruency-based automatic registration algorithm. The phase congruency represents the structural key features such as edges and corners. Instead of using derivatives, the phase congruency defines the feature points in the image where all Fourier components are in phase [29]. The phase congruency is calculated by applying the 2D log Gabor wavelet with multiple scales and orientations [30]. The precision registration involved the following steps:

1. The initial step is to find the key feature points in the RGB-T pair. The phase congruency at scale $k$ and orientation $o$ is used to find the edges and corner points in RGB-T pair. For the image$I(x,y)$, the odd and even components of the 2D log Gabor filter ([${LG}_{ko}^{odd} , {LG}_{ko}^{even}$]) of each scale $k$ and orientation $o$ are applied to calculate the amplitude response ${MR}_{ko}\left(x,y\right)$ of the image [30].

1 $$\left[{ER}_{ko}\left(x,y\right), {OR}_{ko}\left(x,y\right)\right]=[I\left(x,y\right)* {LG}_{ko}^{even} , I\left(x,y\right)*{LG}_{ko}^{odd} ]$$

2 $${MR}_{ko}\left(x,y\right)= \sqrt{{{ER}_{ko}\left(x,y\right)}^{2}+ {{OR}_{ko}\left(x,y\right) }^{2}}$$

The phase congruency of each orientation is calculated by the use of the odd and even responses of the log-Gabor filter [30].

3 $${PC}_{o}\left(x,y\right)= \frac{\sum_{k}{\omega }_{o}\left(x,y\right)*\mathrm{max}(\left(E-{T}_{N}\right), 0)}{\sum_{k}{MR}_{ko}\left(x,y\right)+ \varepsilon }$$

where, the element ${\omega }_{o}\left(x,y\right)$ quantifies the sigmoidal weighting function to penalizes the frequency distribution. The element ${T}_{N}$ is used to effectively wavelet denoising via soft thresholding. The $\mathrm{max}$ function gives the maximum values of enclosed variables. $\varepsilon$ is a small positive real value used to prevent the division by zero. In Eq. (3) $E$ is calculated as:

4 $$E= {ER}_{ko}\left(x,y\right){\overline{\theta }}_{o}^{even}\left(x,y\right) + {OR}_{ko}\left(x,y\right){\overline{\theta }}_{o}^{odd}\left(x,y\right) - \left|{ER}_{ko}\left(x,y\right){\overline{\theta }}_{o}^{odd}\left(x,y\right)- {OR}_{ko}\left(x,y\right){\overline{\theta }}_{o}^{even}\left(x,y\right)\right|$$

where,

5 $${\overline{\theta }}_{o}^{even}\left(x,y\right) =\sum_{k}{ER}_{ko}\left(x,y\right)/{AR}_{o}\left(x,y\right)$$

6 $${\overline{\theta }}_{o}^{odd}\left(x,y\right) =\sum_{k}{OR}_{ko}\left(x,y\right)/{AR}_{o}\left(x,y\right)$$

7 $${AR}_{o}\left(x,y\right)= \sqrt{\sum_{k}{ER}_{ko}\left(x,y\right)+\sum_{k}{OR}_{ko}\left(x,y\right)} + \varepsilon$$

The concept of classical moment analysis equations [31] is applied to the obtained phase congruency maps of each orientation angle $\phi$ as follows:

8 $$p= \sum {(PC\left(\phi \right)\mathrm{cos}\left(\phi \right))}^{2}$$

9 $$q= 2\sum (PC\left(\phi \right)\mathrm{cos}\left(\phi \right))\cdot (PC\left(\phi \right)\mathrm{sin}\left(\phi \right))$$

10 $$r= \sum {(PC\left(\phi \right)\mathrm{sin}\left(\phi \right))}^{2}$$

11 $$M=\frac{1}{2} ( r+p+ \sqrt{{q}^{2}+{\left(p-r\right)}^{2}} )$$

12 $$m=\frac{1}{2} ( r+p- \sqrt{{q}^{2}+{\left(p-r\right)}^{2}} )$$

The $M$ and $m$ are the maximum moment and minimum moment images respectively for an image $I\left(x,y\right).$ The FAST [32] algorithm is applied on the $M$ and $m$ to extract the edges and corners keypoints in image $I(x,y)$.

2. Feature descriptors are used to distinguish feature points. The feature descriptor quantified the feature points with the neighbourhood information. The calculated log-Gabor responses are utilized to find the feature descriptor. For each orientation $o$, the sum of the amplitude response $MR (x,y)$ (using Eq. (2)) at each scale $k$ is calculated and they are stacked for each orientation $o$ to form an array $\{{{MR}_{o}^{k}\left(x,y\right)\}}_{1}^{{N}_{o}}$, where ${N}_{o}$ represents the total number of orientations. The maximum value of $\{{{MR}_{o}^{k}\left(x,y\right)\}}_{1}^{{N}_{o}}$ at each pixel location $(x,y)$ and its index values $i$ are utilized to build a histogram for feature description similar to SIFT [33].
3. After locating the feature points and corresponding point descriptors in both images, the sum of square differences is used to perform feature matching. When the nearest neighbour distance ratio is met, the feature points of two images are matched. The matched feature points are used to calculate the geometrical affine transformation matrix using fast sample consensus (FSC) [34].

### ROI selection and tracking

The fundamental concept of this work is to use the RGB image sequences for ROI selection and tracking, then by applying the obtained transformation matrix through registration, to conduct linear mapping of the ROI in the thermal image sequences. The mapped ROI in the thermal images is then used to extract the respiratory signal. The RGB image sequences have more detailed information compared to the thermal images and have a large database and pre-trained models for the detection of facial features. Haarcascade classifiers [35] for nose detection, multi-task cascaded convolution neural network (MTCNN) [36] and, more recently, the YOLO5Face detection [37] model are mostly used for the detection of faces and facial keypoints. The YOLO5Face detection model is designed by the YOLOv5 object detector [38]. The five key facial landmark regression introduced in the YOLOv5 model with the Wing loss function. Additionally, the key modification in stem block structure and the block in the architecture were introduced to make it for the face and face landmark detection. It was not only achieved high accuracy than the state-of-the-art techniques, but also perform faster [37]. The YOLO5Face model can accurately detect the centre of the left and right eye, the tip of the nose, and the right and left mouth corners. The ROI defining the nostril region consists of the rectangular bounding box defined by corner (bx,by), width w and height h. Assuming that (nx,ny), (mlx,mly), and (mrx,nry) are the coordinates of the tip of the nose, the left corner of the mouth, and the right corner of the mouth, respectively. The ROI for RR monitoring is defined as bellow:

13 $$ROI=\left[bx ,by , w , h\right]=[nx-\omega , ny-\eta , 2\cdot \omega , 3\cdot \eta ]$$

where,

14 $$\eta =round\left(\frac{\sqrt{{\left(mlx-nx\right)}^{2}+ {\left(mly-ny\right)}^{2}}}{4}\right)$$

$$\omega =round\left(\frac{\left|mlx-mrx\right|}{2}\right)$$

The tracking approach utilizes either the manual selection of objects in the initial frame and performing the tracking in subsequent frames or combining the detector model with the tracking model to perform automatic tracking, which is generally called the "tacking-by-detection (TD)" approach [39]. The TD-based method is simple to implement and performs efficiently in terms of object variation, scene variation, and the number of targets. In this work, the deep learning-based tracking by detection approach is used, which includes the YOLO5Face detector [37] and the dlib correlation tracker [40] to perform the automatic ROI selection in more challenging conditions, e.g., rapid movement, multiple people, and high dynamic background. Also, it handles the deviation of objects from the FOV or occlusions by reinitialization of the detectors. As illustrated in Fig. 1, the deep learning-based TD technique used a selection operator to associate detection with tracked frames.

The functioning of the deep learning-based TD approach for ROI detection and tracking has been explained in Algorithm 1. The main work of the selection operator is to enable learning of the tracking model when tracking fails. The status of tracking and detection is defined by ${t}_{s}$ and ${d}_{s}$, respectively. At the initial frame of the video, the detection is performed, and the tracking is initialized with the selected ROI. After that, the function of selection operator is performed in successive frames as follows:

1. When both ${t}_{s}$ and ${d}_{s}$ are True, tracking will be enable in the successive frame,
2. When one or both of ${t}_{s}$ and ${d}_{s}$ are False, the tracking will disable, and detection will be performed for the ROI detection.

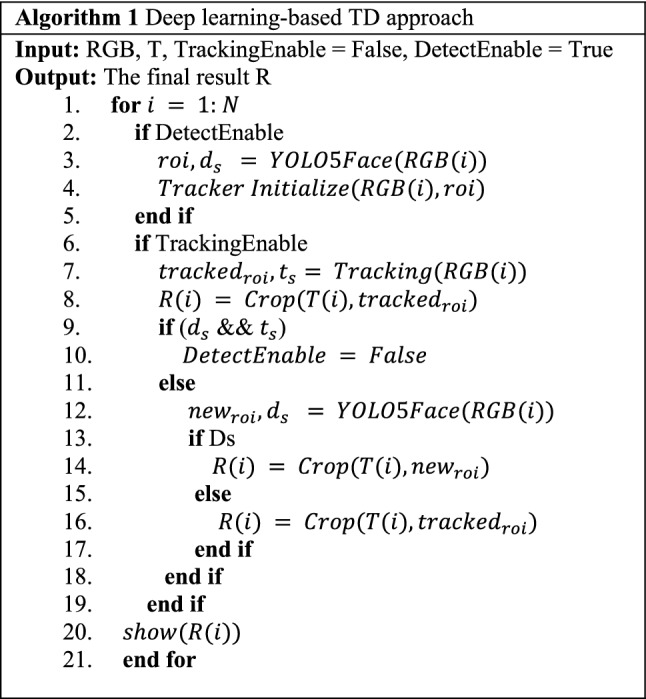


### Extraction of respiratory signal and RR

The cropped image $R$ obtained by the deep learning-based TD framework in the RGB frame is linearly mapped to obtain the ROI in the thermal image. The linear mapping is obtained by applying the transformation matrix obtained through the RGB-T image registration. The mapped ROI ${IRT}_{ROI}$ has the respiratory signal information, and the respiratory signal is obtained by the averaging of the pixels value in the ${IRT}_{ROI}$. The raw respiratory signal $\overline{rs }$ in each frame $i$ of the video sequence is calculated as:

15 $$\overline{rs }\left(i\right)= \frac{1}{W\times H}\sum_{x=0}^{W-1}\sum_{y=0}^{H-1}{IRT}_{ROI}(x,y,i)$$

where, the ${IRT}_{ROI}$ is the mapped ROI in the thermal image. The width and height of the mapped ROI are denoted by $W$ and $H$, respectively. To extract the smoothed respiration signal, filtering approaches are applied to the normalized raw respiratory signal. First, the unwanted spikes are filtered out by a Hampel filter [41], secondly, a moving average filter is applied to smooth out abrupt changes. Lastly, we used a Butterworth bandpass filter of 2nd order with a configuration of 3 dB low pass and high pass cut off range [0.1, 0.85] for the healthy adults and [0.5, 1.5] for newborn infants, respectively. The respiratory rate is obtained by applying the chirp Z transformation (CZT) [42] on the smoothed respiratory signal. In clinical practice, the physicians and nursing staffs count the breathing cycle either in 30 or 60 s for the RR measurements [43]. To analyse and calculate the rate, the window of 30 s with a sliding step of 20 s has been used in this work.

## Experimental setup and protocol

The developed algorithm has been applied first to healthy volunteers to validate the feasibility of the work. The data of healthy adults were collected to evaluate the human breathing rate in a room environment. The newborn data was obtained from the neonatal intensive care unit in the presence of health experts and nursing staff. The camera configuration used was a dual camera setup as shown in Table 1.

**Table 1:** The configuration and specifications of the cameras used in the experiments

Modality	Name	Type	Fps	Resolution	Thermal sensitivity	Reference
Infrared thermography	FLIR-E60	LWIR	30	320 × 240	0.05 °C at 30	FLIR Systems Inc
Visible	C922 webcam	Color	30	960 × 720	–	Logitech International S.A

### Study in healthy adults

The human data is collected in three protocol settings to see the efficiency of the proposed approach in more challenging cases like high movement, talking, and dynamic breathing patterns. The study included the 14 healthy adults with an average age of 29.7 ± 2.8. The dual camera setup was mounted on a tripod and was about 180 cm away from the subjects. The data is collected mainly in three modes, Mode A, B, and C. In Mode A, the person was seated on a chair in a comfortable, steady position and was told to breathe at their own pace for a time period of 1 min 20 s. Mode A setting is to see the efficacy in clinical setting scenario. In Mode B, persons were allowed to move and talk during the breathing recording. However, the person was restricted to be seated on the chair and not performing any movement beyond the FOV. Mode B is attempting to simulate practical scenarios such as measurements in social gatherings, quarantine centers, and airport screening. The duration of recording was the same as in Mode A. In Mode C, the healthy adults have been told to breathe in varying orders, such as normal, fast, and stop for a while during the recording. The recording of Mode C was 3 min.

The ground truth respiratory signal was collected simultaneously with the RGB-T data recording in each mode utilizing the Go Direct® respiration belt [44]. The belt measures the chest or abdominal movement during respiration with 0.01 N force resolution and the data is recorded at 10 samples per second.

### Study in neonates

To study the feasibility of the proposed method on newborn infants in a clinical setting, ten RGB-T video data sets of clinically stable newborn infants were obtained. The camera setup and videos were recorded at Government Medical College Hospital (GMCH), Chandigarh and were approved by the Institute Ethics Committee of GMCH (Approval No: GMCH/IEC/2020/522/87). The dual-camera setup was placed approximately 1 m away from the subjects. The room temperature and humidity were set according to the acceptable level of the intensive care unit. The study excluded neonates who needed respiratory support, had dyspnea or tachypnea, or had unstable vital parameters, an elevated temperature, or any congenital anomaly.

### Evaluation metrics

The proposed contactless breathing monitoring method is validated with the contact base reference method. To evaluate the effective performance and to analyse the error, the average absolute error (AAE) and the standard deviation error (SDE) have been calculated between the proposed and contact-based methods. ${BR}_{m}\left(k\right)$ and ${BR}_{r}\left(k\right)$ are the respiratory rate of proposed contactless approach and contact-based approach for each measurement ‘*k*’, respectively, and $N$ is the total number of measurements for each subject, the AAE and SDE are calculated as follows:

16 $$AAE= \frac{1}{N} \sum_{i=1}^{N}AE\, ; where, AE= \left|{BR}_{m}(k)-{BR}_{r}(k)\right|$$

17 $$SDE= \sqrt{\frac{1}{N-1}\sum_{k=1}^{N}{(AE\left(k\right)-AAE)}^{2}}$$

The Bland–Altman analysis and correlation plot are utilised to graphically and statistically demonstrate the proof of agreement between the proposed technique and the reference method. The effectiveness of the ROI selection by using the deep learning-based TD approach is evaluated by the ROI success rate. The ROI success rate is defined as follows:

18 $$ROI\,success\,rate= \frac{ROI\,detected }{number\,of\,frames } \times 100$$

## Result

**Fig. 2:**
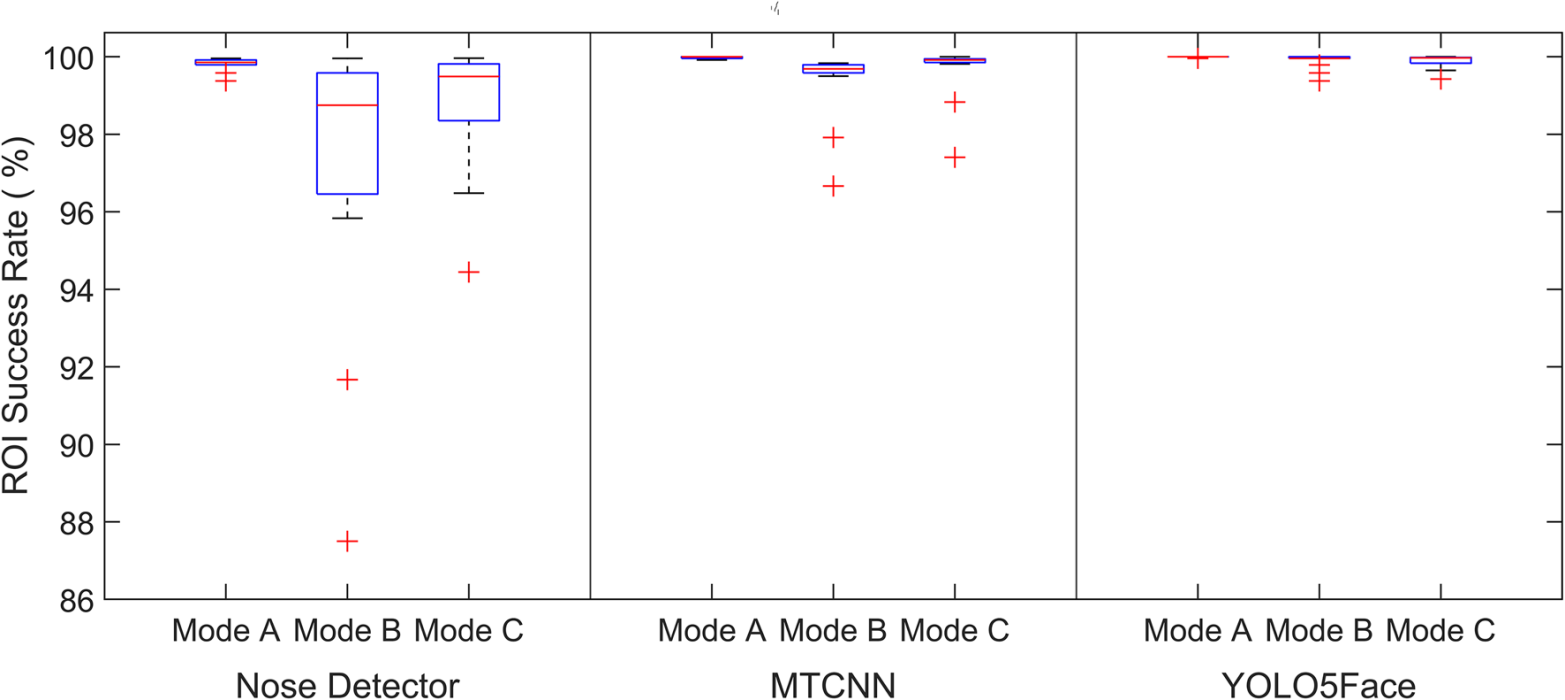
The boxplot of the ROI success rate of Nose detector, MTCNN, and YOLO5Face models for healthy adults in different modes

**Fig. 3:**
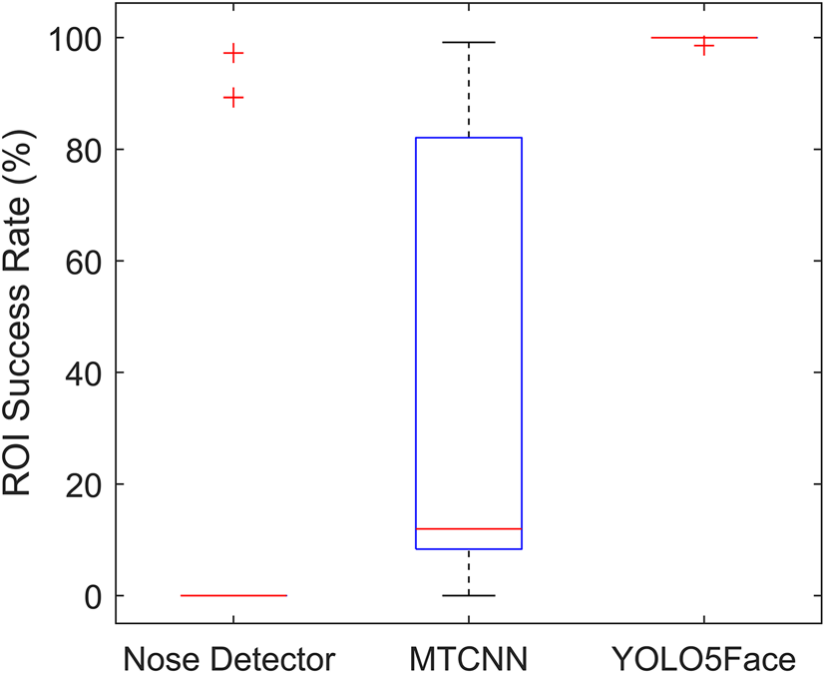
The boxplot of the ROI success rate of Nose detector, MTCNN, and YOLO5Face model for newborn data

Automatic facial landmark detection is crucial for the successful extraction of RR. Instead of only detection, it is also important to note how accurately a landmark is detected in successive frames. In the proposed method, the YOLO5Face detection model has been used because of its fast and efficient accuracy in a wide range of databases. Here, the performance of YOLO5Face detection is compared to that of the current state-of-the-art Nose detector and MTCNN in the deep learning-based TD method for ROI detection. Figure 2 shows a boxplot comparison of ROI success rates for healthy adults in various modes. In Mode A, the average ROI success rates for nose detection, MTCNN, and YOLO5Face were 99.82%, 99.98%, and 99.99%, respectively. For Mode B, the obtained values were 93.75%, 99.37%, and 99.90%, respectively, and for Mode C, the values were 98.85%, 99.66%, and 99.88%, respectively. It has been observed that the YOLO5Face model has a higher average success rate than the other state-of-the-art models. Most importantly, the YOLO5Face model successfully performs ROI detection in the newborn dataset. Figure 3 shows the boxplot analysis of different models for the newborn dataset. The average of the ROI success rates in the newborn dataset for Nose detection, MTCNN, and YOLO5Face model are 18.65%, 32.96%, and 99.86%, respectively.

**Fig. 4:**
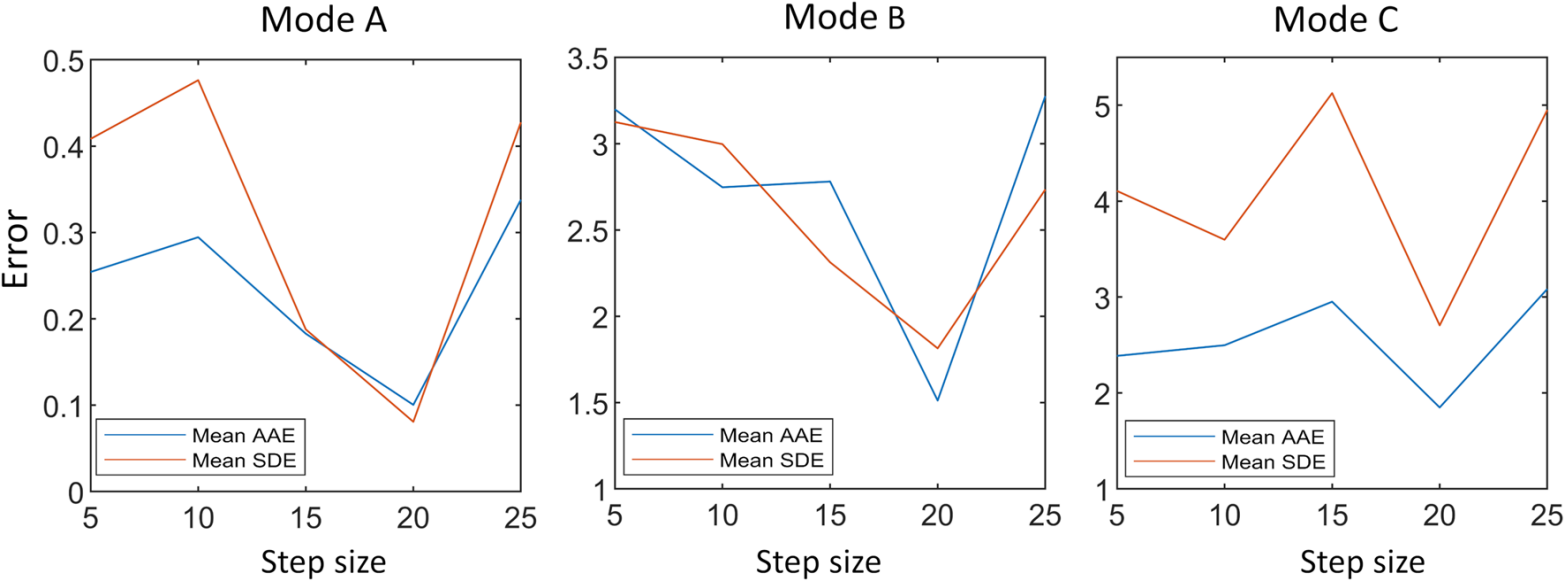
The mean AAE and mean SDE values for different values of window step size for healthy adults’ study

We evaluated the performance of various window step sizes for assessing the respiratory rate in the healthy adults’ data. Figure 4 demonstrates the mean AAE and mean SDE in each mode of data collection for healthy adults. It has been observed that for window step size 20, the mean AAE and mean SDE were less than the lower and higher values of window step size. In the case of small step sizes, mostly the movements affect the results, whereas if the step size is larger than 20, most of the signal information is overlooked during the evaluation.

**Fig. 5:**
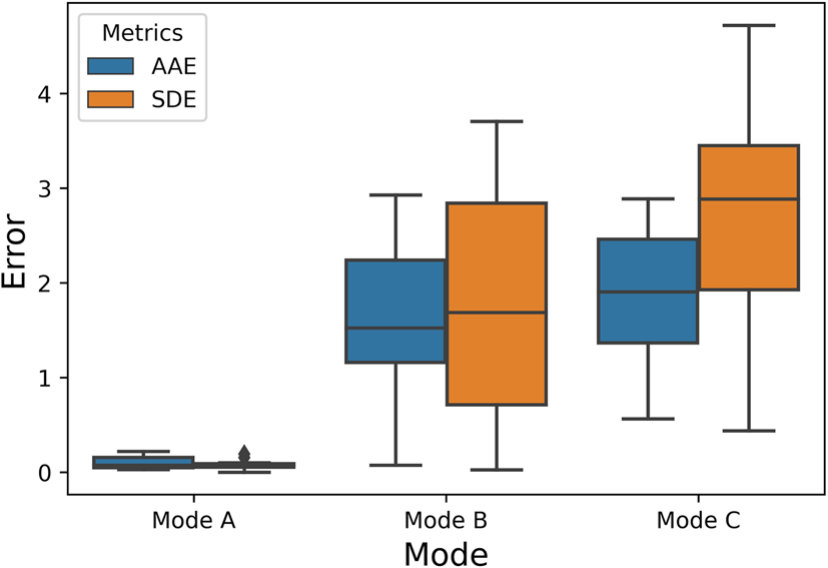
The boxplot of AAE and SDE between the proposed approach and the contact-based reference method for different modes in healthy adult study

Figure 5 shows the boxplot of AAE and SDE in different modes for healthy adults. The mean of AAE and SDE was 0.1004 ± 0.0608 and 0.0808 for Mode A. Moreover, the estimated mean AAE and SDE between the proposed and contact-based methods were 1.5115 ± 0.9058 and 1.8138 for Mode B, and they were 1.8485 ± 0.8079 and 2.7048 for Mode C, respectively. In the obtain results, 100%, 71.40%, and 57.14% of the absolute error results were below than the 2 breaths/minute.

**Fig. 6:**
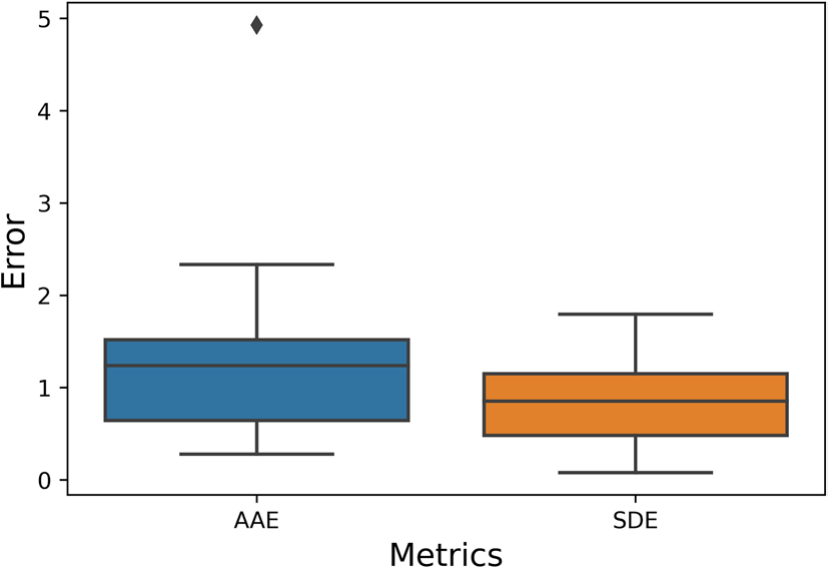
The boxplot of AAE and SDE between the proposed approach and the contact-based reference approach for neonate study

**Fig. 7:**
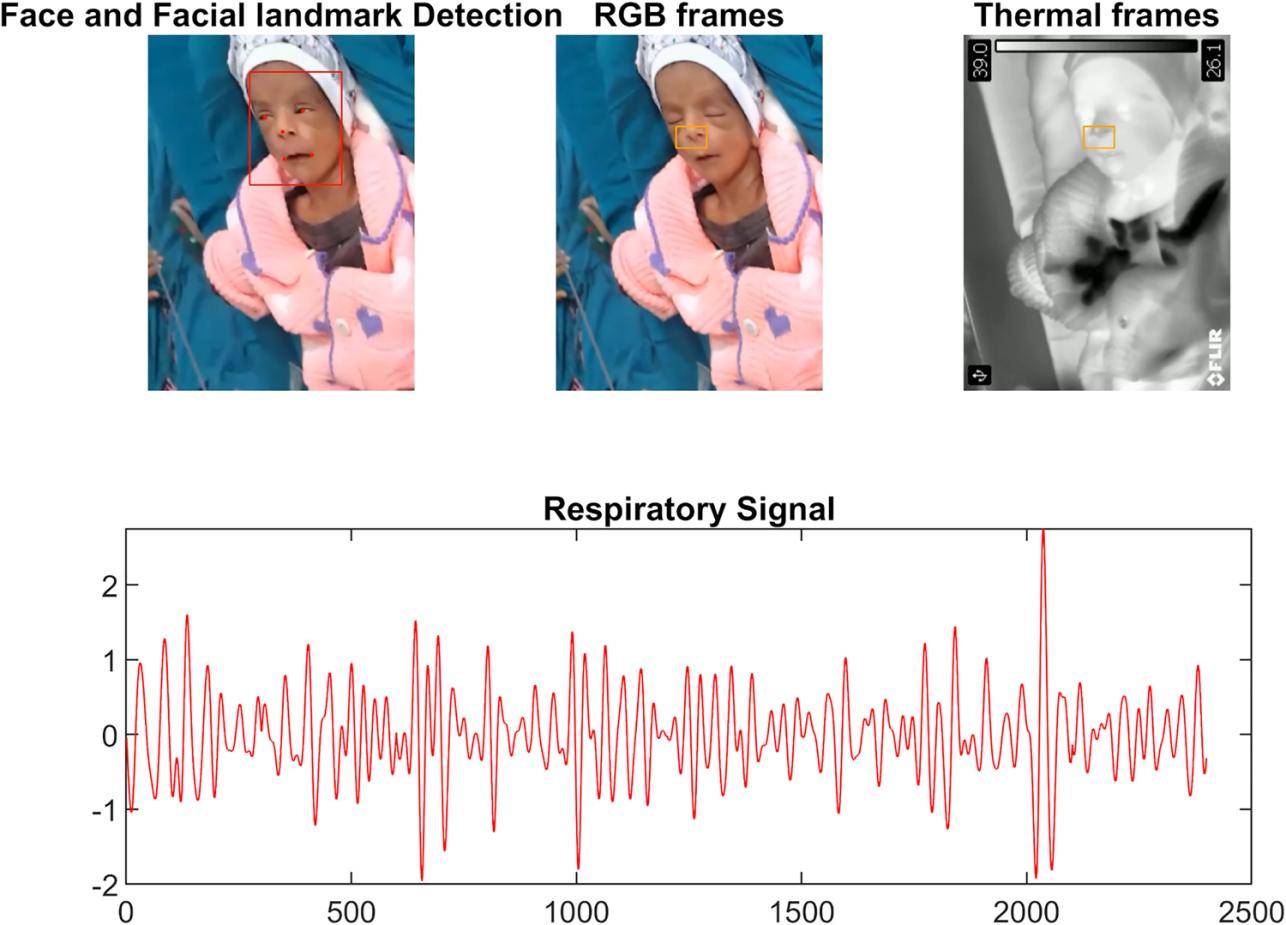
The visualization of the respiratory signal extraction using the proposed contactless method. It can be found in Video A depicting the functionality of the proposed method

In the case of neonate data, the boxplots of error metrics are shown in Fig. 6. The observed mean AAE and SDE were 1.4861 ± 1.3567 and 0.8591, respectively. Figure 7 depicts the visualisation of facial landmark detection and tracking using the deep learning-based TD approach and the respiratory signal extracted by the mapped ROI in the thermal image. The visualisation is shown in Video A (see Supplementary files) to represent the working functionality of the proposed non-contact-based method.

**Fig. 8:**
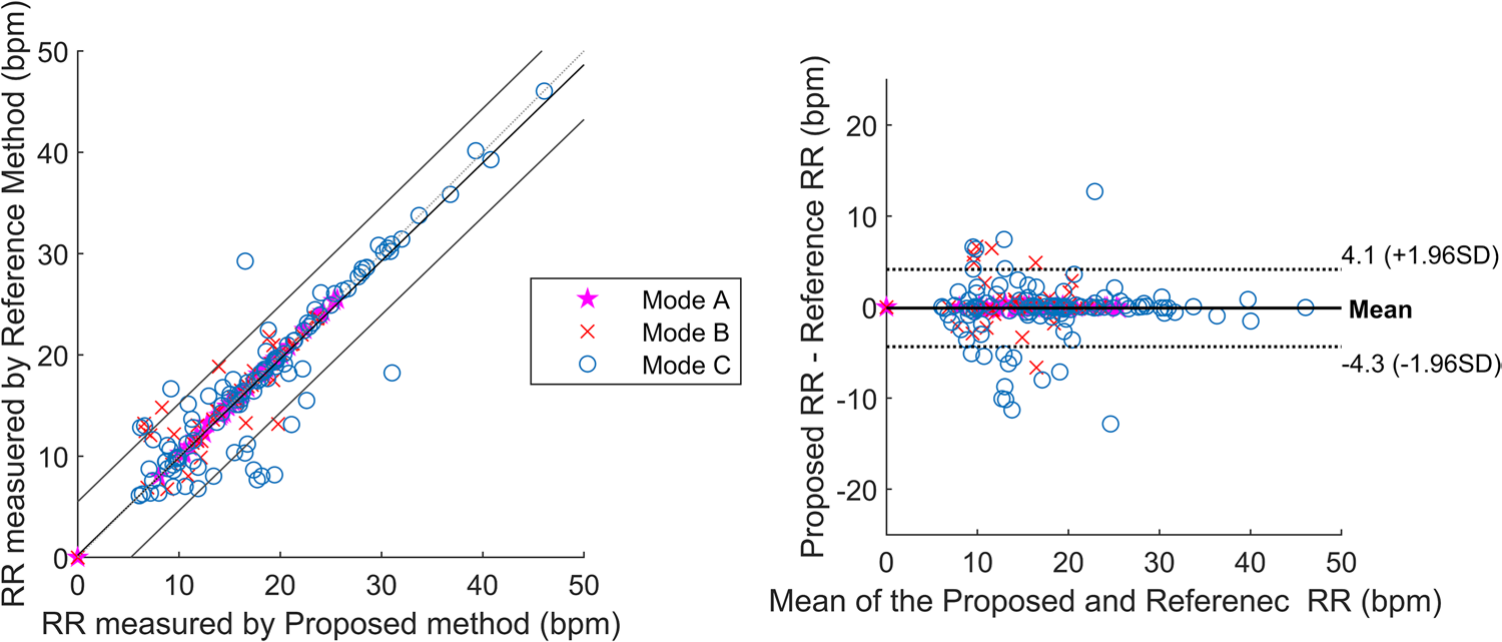
The linear correlation plot (left) and Bland–Altman plot (right) for the different mode of health adult study. Total 196 RR measurement data points. The different modes are represented by different colours and symbols

**Fig. 9:**
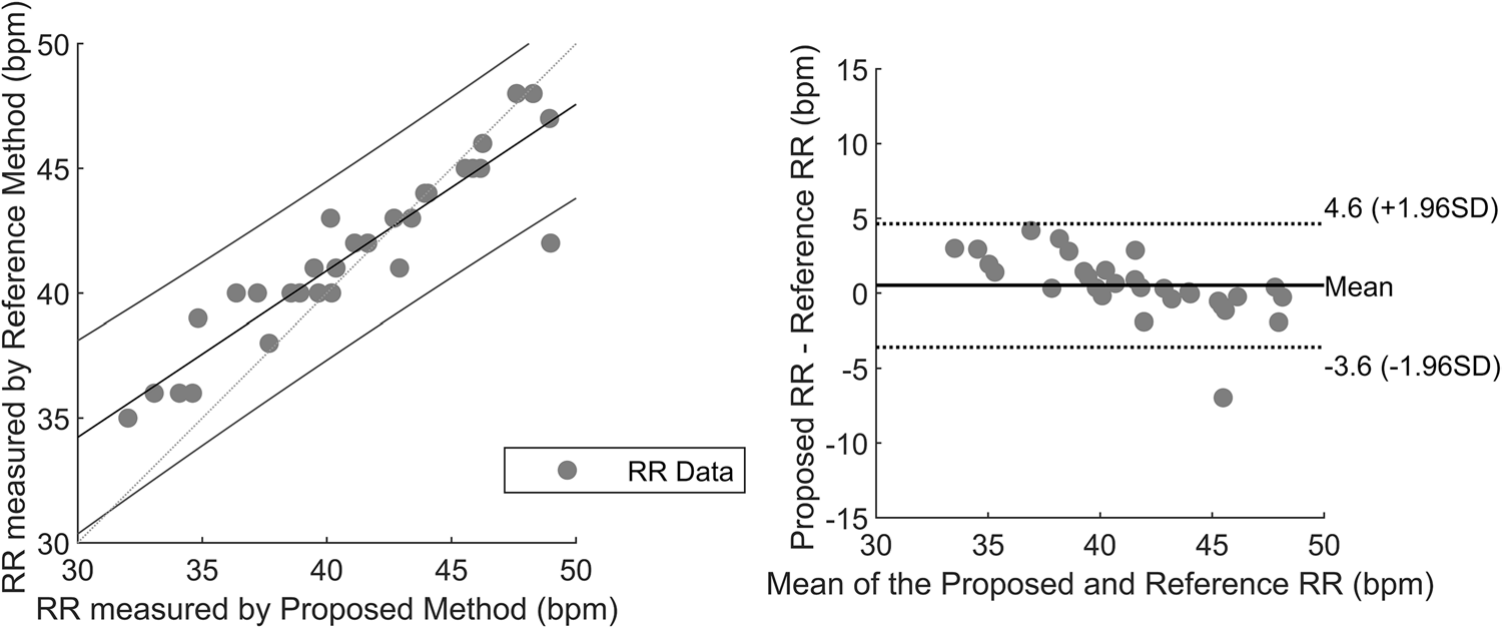
The linear correlation plot (left) and Bland–Altman plot (right) for the newborn infant study. Total 30 RR measurements data points

The Bland Altman and linear correlation plots for aggregating all of the mode's RR measurement data for healthy persons are displayed in Fig. 8. The results indicate a strong correlation between the proposed contactless approach and contact based approach. By observing the Bland Altman plot, the mean difference between the proposed contactless and contact-based approaches for all the RR measurements was − 0.11 breaths/minute with the 95% limit of agreement range from − 4.3 breaths/minute to 4.1 breaths/minute. Figure 9 depicts the Bland–Altman plot for the neonate study. The analysis shows that the mean difference between proposed RR and reference RR was 0.51 breaths/minute and the limit of agreements ranged from -3.6 breaths/minute to 4.6 breaths/minute.

## Discussion

The experimental results indicate that the proposed non-contact method using thermal and visible imaging performs better for respiratory rate monitoring in both stable and challenging conditions like rapid head movement, talking, and variable breathing patterns in healthy adults. More importantly, the selection of ROI is automatic, with an ROI success rate of more than 99% in each mode of the dataset. The YOLO5Face detection model outperforms other state-of-the-art methods in each mode and efficiently works in the case of newborn infants' nostril area detection. The automatic ROI selection was the limitation of existing work in the literature because thermal imaging has less information and poor resolution as discussed in [22, 45, 46]. The cost is the prime factor for the high-resolution camera. The average absolute error and the correlation coefficient in the stable mode are approximately 0.1 bpm and 0.9994, respectively. The findings are similar to a study conducted by Pereira et al. [47] when they used a high spatial resolution infrared camera with a spatial resolution of 1024 × 768 pixels for respiratory rate detection. The methodology of using the RGB spectrum along with the thermal spectral image sequences eliminates the need of utilizing of thermal images feature for ROI selection and tracking. The RGB spectrum is used for the ROI detection and the mapped ROI in the thermal image sequence is used to extract the respiratory signal and respiratory rate. In the proposed method, the deep learning-based TD approach performs the ROI detection and tracking, and it reinitializes the detection if the tracking fails due to the presence of an obstacle or out of the FOV condition. During the rapid head movement and talking, the proposed method performs better as detection is reinitialized when the obstruction is removed and the respiratory signal is collected again. The results demonstrated that for Mode B and Mode C scenarios, the average error is approx. 1.5 bpm and 1.8 bpm, respectively, which is under the error of 2 bpm for the challenging conditions such as motion and talking, and has better agreement with the reference method than the study performed by Chauvin et al. [48]., which included the breathing detection during talking and paddling.

After the method was successfully validated on healthy adults, the proposed method was also tested on newborn data in a clinical setting. The results demonstrated the mean of AAE between the proposed and reference methods is approximately 1.5, about 80% are the relevant estimation i.e. error less than 2 breaths/minute. The correlation plot shown in Fig. 9 shows a significant correlation with the reference method, and the calculated correlation coefficient is 0.9244. The proposed approach detects the ROI automatically and employs efficient tracking to record the continuous respiration rate, which was the limitation of the study conducted on 8 neonates by Abbas et al. [22]. The integration of RGB with thermal image sequences includes the characteristics of using standard thermal resolution with better results than the work proposed by Pereira et al. [24] using a high-resolution camera (VarioCAM® HD head 820S/30 mm, InfraTec). Another study by Pereira et al. [25] used the ‘black-box’ approach by choosing the best ROI with high signal quality index, but the results were mostly affected by newborn infants’ motion. Overall, the mean AAE and Bland Altman plot analysis were comparable with the studies by Pereira et al. [25] and our proposed method showed less spread of error in the range of − 4.3 breaths/minute to 4.1 breaths/minute in the Bland Altman plot analysis. However, in overall neonate dataset, two infants N_4 and N_10 exhibited head motion during recording, still methodology successively records the RR continuously and the resultant AAE values were 4.9280 and 2.3350, respectively. The infant N_4 showed a failure of ROI detection and tracking during recording because of head rotation. However, after some frames, when the infant's head returns to the camera’s FOV, the deep learning-based TD approach automatically detects and tracks the ROI for respiratory signal extraction.

**Fig. 10:**
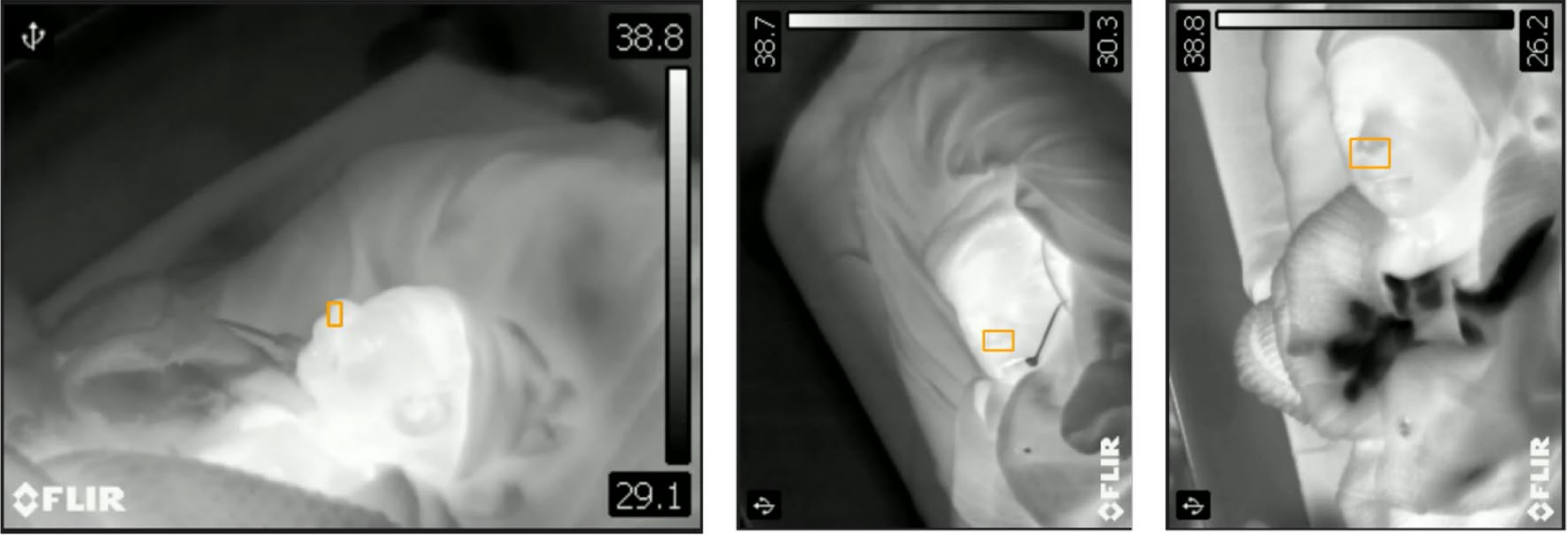
The sample images recorded by the thermal camera showing the challenges associated with the newborn’s RR measurements. The babies, mostly covered by the blanket to keep them warm, have less anatomical area for feature extraction

There are several challenges associated with the data collection of newborn infants in the clinical setting. One of the primary challenges is the stability of the babies. Normally, they move their heads, hands, and legs during the awake period. Babies in open beds are the major population, and they are usually covered by a blanket to keep them warm as shown in Fig. 10. In such a situation, the detection of movement and feature extraction in thermal imaging is a challenging task. Also, the method of respiration detection based on skin segmentation [49] and motion extraction from the camera [25] has limitations when most of the baby’s body is covered with a blanket. Our study provides the methodology of respiration signal extraction from the temperature variation within the surface of the nostril area in the face, which is mostly uncovered except when other contact-based devices are not attached to the baby’s face. The RGB images are used to find the ROI and tracking as RGB images have a large extent of information in comparison to thermal images and have pre-trained models to extract the feature to a deep extent successfully. The deep learning-based TD approach automatically detects the nostril area as an ROI in the RGB spectrum, overcomes the problem of high movements and less anatomical area for feature detection in the FOV of cameras, and the linearly mapped ROI in thermal image sequences extracts the respiratory signal efficiently. The head rotation observed in the infant N_10 caused ROI detection failure in some frames, but the deep learning-based TD approach was able to continue tracking the ROI when the head is in the FOV of the camera. Although, during this interval of time, the value of RR is affected and it was also observed as an outlier in the boxplot and Bland Altman plot shown in Figs. 6 and 9. The success rate of YOLO5Face is higher in healthy adults and successfully works in newborn cases in comparison to other models, making it a more prominent choice for newborn respiration detection.

One of the problems with respiration that occurs in the newborn is apnea, and the accurate diagnosis of clinically significant episodes of infant apnea is a clinical necessity. However, the existing chest impedance (CI)-based monitoring used in clinical practise has major issues with mistaking the cardiac signal for breathing during apnea [50]. The proposed thermal imaging-based approach detects the respiration signal by measuring the nasal area temperatures, which directly depend on the process of respiration, and the cessation of breathing might be detected. Further efforts will be focusing mostly on utilizing and improving our method for detection of apnea. During the initial few days, the incubator temperature is closer to the body temperature. However, the proposed approach may easily detect if there is a temperature variation in the nostril area. Thermal camera has a thermal sensitivity of 0.05 °C at 30 °C environment temperature. Further studies are needed to test the usefulness of the proposed method in this special situation.

In NICU, sick preterm neonates are frequently managed with CPAP or a high-flow nasal cannula [51]. In this scenario, challenges in the non-contact monitoring include altered temperature due to gases at the nasal interface and the area around the nose being covered with adhesives to fix the nasal respiratory interface. However, the temperature of the inhaled gases should not interfere with the monitoring, as the inhaled gases are not cold but heated and humidified. We believe that in this special situation, some alteration in the modelling may be needed due to a change in the airflow pattern and a narrower difference between the temperatures of the inhaled and exhaled gases. Again, further studies are needed in this special situation.

## Conclusion

Non-contact respiratory rate monitoring is important and should be used in the intensive care unit because it is harmless, passive and does not require any connecting wires. This paper introduced the methodology for non-contact RR monitoring in noenates. The integration of RGB spectrum data enables the use of an efficient deep learning model for ROI selection and tracking. The detected ROI in the RGB spectrum is linearly mapped to find the correspondence ROI in thermal imaging for respiratory rate extraction. The deep learning-based TD approach reinitializes the detection in the case of failed tracking due to the presence of an obstacle or the out of FOV scenario.

Initially, the algorithm was validated by the contact-based respiration belt with high accuracy on the healthy adult data collected in the lab settings. Subsequently, the proposed method was validated on newborn infant data recorded in a clinical setting. The results were promising, and the average absolute errors between the estimated and reference methods were less than 2 bpm. In conclusion, the proposed method robustly extracts the RR by utilizing RGB-T image sequences not only in a lab setting but also in a clinical setting for newborn infants and is a clinically relevant alternative to the contact-based method.

## Supplementary Information

Below is the link to the electronic supplementary material.

Video A: it is the visualization of the extraction of respiratory rate using proposed methodology. Supplementary file1 (MP4 16527 KB)
